# Potential role of dengue virus, chikungunya virus and Zika virus in neurological diseases

**DOI:** 10.1590/0074-02760170538

**Published:** 2018-10-29

**Authors:** Marcelo Adriano da Cunha e Silva Vieira, Carlos Henrique Nery Costa, Alexandre da Costa Linhares, Amariles de Sousa Borba, Daniele Freitas Henriques, Eliana Vieira Pinto da Silva, Fernando Neto Tavares, Francisca Miriane de Araújo Batista, Herlon Clístenes Lima Guimarães, Lívia Carício Martins, Talita Antônia Furtado Monteiro, Ana Cecília Ribeiro Cruz, Raimunda do Socorro da Silva Azevedo, Pedro Fernando da Costa Vasconcelos

**Affiliations:** 1 Secretaria de Estado da Saúde do Piauí, Instituto de Doenças Tropicais Natan Portella, Departamento de Neurologia, Teresina, PI, Brasil; 2 Fundação Municipal de Saúde de Teresina, Diretoria de Vigilância em Saúde, Teresina, PI, Brasil; 3 Instituto Evandro Chagas, Programa de Pós-Graduação em Virologia, Ananindeua, PA, Brasil; 4 Secretaria de Estado da Saúde do Piauí, Instituto de Doenças Tropicais Natan Portella, Departamento de Infectologia, Teresina, PI, Brasil; 5 Instituto Evandro Chagas, Seção de Virologia Geral, Ananindeua, PA, Brasil; 6 Instituto Evandro Chagas, Seção de Arbovirologia e Febres Hemorrágicas, Ananindeua, PA, Brasil; 7 Secretaria de Estado da Saúde do Piauí, Diretoria da Unidade de Vigilância e Assistência à Saúde, Teresina, PI, Brasil

**Keywords:** chikungunya virus, dengue virus, encephalitis, Guillain-Barré syndrome, transverse myelitis, Zika virus

## Abstract

This study showed that laboratory markers of recent infection by dengue, Zika or chikungunya arboviruses were detected in the biological samples of approximately one-third of patients with encephalitis, myelitis, encephalomyelitis or Guillain-Barré syndrome, in a surveillance programme in Piauí state, Brazil, between 2015-2016. Fever and myalgia had been associated with these cases. Since in non-tropical countries most infections or parainfectious diseases associated with the nervous system are attributed to herpesviruses, enteroviruses, and *Campylobacter jejuni*, the present findings indicate that in tropical countries, arboviruses may now play a more important role and reinforce the need for their surveillance and systematic investigation in the tropics.

Arboviruses are responsible for a wide spectrum of clinical syndromes, ranging from mild to severe febrile illnesses, haemorrhagic fever or neuroinvasive diseases.^1^ In tropical regions, infections by dengue virus (DENV) are associated with meningitis, encephalitis, myelitis and polyradiculoneuritis.^2^ In addition to causing acute febrile polyarthralgia and prolonged arthritis, infection with chikungunya virus (CHIKV) can evolve into severe neurologic complications, such as acute flaccid paralysis and encephalitis.^3^

The recent Zika virus (ZIKV) infection epidemic in the Americas, recognised in Brazil in 2015, was associated with an increase in the number of microcephaly and Guillain-Barré syndrome (GBS) cases.^4^ Thereafter, atypical cases of ZIKV were also described as associated with severe neurological impairment, including encephalitis, meningitis, acute transverse myelitis (ATM), and acute disseminated encephalomyelitis (ADEM), as well as with cases of death in individuals with autoimmune diseases.^5^ ^,^ ^6^

The first human encephalitis case caused by West Nile virus (WNV) in Brazil was detected in Piauí state in 2014.^7^ Since then, the Piauí state department of epidemiology has instituted a surveillance programme for neurological disease. Cases of aseptic meningitis, encephalitis, ATM and GBS have been submitted for epidemiological and laboratory investigation, which allows monitoring the occurrence of these disorders.^8^

The first records of Zika fever and chikungunya fever in Piauí state took place in 2015, when over 7,000 suspicious cases of dengue were reported.^8^ In response to the preliminary information that ZIKV infections could be related to the increase in acute neurological syndromes, the Brazilian Ministry of Health (MoH) proposed a surveillance protocol for neurological manifestations due to viral infections, in November 2015.^9^ In accordance with this protocol, laboratory tests for DENV, ZIKV, and CHIKV should be performed in encephalitis, ATM, ADEM and GBS cases reported in Piauí state.

The goal of this study was to assess the presence of clinical and laboratory indicators of recent DENV, ZIKV and CHIKV infections in acute encephalitis, ATM, ADEM and GBS cases.

## MATERIALS AND METHODS

The MoH surveillance protocol for neurological manifestations due to viral infections recommended the selection of a single reference hospital for diagnostic and case investigation in Piauí state, but the state epidemiology department chose to maintain the surveillance of neuroinvasive syndromes decentralised and with higher sensitivity, akin to the strategy used to detect new WNV infection cases. Therefore, all cases of ADEM, ATM, GBS and encephalitis, regardless of fever, rash, myalgia or arthralgia, were reported and investigated. Physicians in Piauí state were informed of the surveillance protocol by presentations at clinical meetings at selected hospitals, by correspondence with infectious and neurological disease physicians, and through local health departments. Patients were referred to the study by physicians or by infection control practitioners. Accordingly, encephalitis, ATM, ADEM and GBS cases were identified through daily verification of the diagnostic hypothesis listed in the medical records of the hospitalised patients - performed by hospital epidemiological surveillance teams (active surveillance), and by spontaneous notifications by assistant medical teams (passive surveillance). Five tertiary care hospitals in Piauí state were considered sentinel units, but all other hospitals were also able to notify and investigate cases. Thereafter, the state epidemiology department was notified of suspected cases by infection control practitioners and health professionals from 11 hospitals in Piauí state and through ongoing state-based surveillance for neurological manifestations due to WNV and other arboviral infections. The cases of encephalitis, myelitis and encephalomyelitis attributed to central nervous system (CNS) bacterial, mycobacterial, fungal or parasitic infections or to autoimmune diseases were not included in the surveillance protocol.^9^

Serum and cerebrospinal fluid (CSF) samples taken during the acute phase were subjected to quantitative reverse transcription real-time polymerase chain reaction (RT-qPCR) for genome detection of DENV, ZIKV and CHIKV.^10^ ^,^ ^11^ ^,^ ^12^ ^,^ ^13^ Enzyme-linked immunosorbent assays (ELISAs) were performed to detect IgM antibodies against DENV, ZIKV and CHIKV in paired sera and CSF.^14^ ^,^ ^15^ For the differential diagnosis, CSF samples from patients with CNS damage (encephalitis, ATM and ADEM) were tested by polymerase chain reaction (PCR) for herpesviruses and by reverse transcription polymerase chain reaction (RT-PCR) for enteroviruses.^16^ ^,^ ^17^ For GBS cases, serum samples were also tested for *Campylobacter jejuni* IgM antibodies by ELISA (Serion ELISA Classic, Institute Virion/Serion, Wurzburg, Germany).

As part of the strategy to detect new cases of WNV infection that was implemented in 2014, serological and molecular tests for WNV were also performed on the study samples. A panel of hemagglutination inhibition tests in paired sera and CSF samples was used to detect antibodies against other *Flavivirus* (Saint Louis encephalitis virus, Ilheus virus and Rocio virus), *Alphavirus* (Eastern equine encephalitis virus and Western equine encephalitis virus), and *Orthobunyavirus* (Oropouche virus), as previously described, as a laboratory screening procedure to broaden the diagnostic protocol for nervous system infections.^18^

Following clinical, radiological, electrophysiological and histopathological parameters, neurological cases were classified according to the Brighton collaboration diagnostic certainty levels. The cases attributed to arboviral infections were classified according to the Centers for Disease Control and Prevention (CDC) neuroinvasive arboviral infection case definitions, following clinical and laboratory criteria.^19^ The association between the presence of clinical and laboratory findings suggestive of arbovirus infection and the aetiology of neurological damage was assessed by Fischer’s exact test (p < 0.05).

*Ethics committee approval* - This study was approved by Piauí State University Ethics Committee under the register no. 2.146.280/2017.

## RESULTS

In the 12-month period from November 2015 to October 2016, there were 74 reports of neurological syndrome cases possibly related to viral infection: encephalitis (35), GBS (21), transverse myelitis (12) and encephalomyelitis (6). The 74 reported cases were classified (according to Brighton collaboration level of diagnostic certainty), as follows: 21% reached level 1, 51% reached level 2, and 28% reached level 3.

**TABLE I:** Serological and molecular tests that defined the etiological diagnosis of 74 patients with acute neurological impairment^*a*^

Etiology	nº (%)	Laboratory test^*b*^
Chikungunya virus	13 (17)	CSF IgM (4), CSF RT-qPCR (1), and serum IgM (8)
Dengue virus	8 (11)	CSF RT-qPCR (2) and serum IgM (6)
Herpes simplex virus	7 (9)	CSF PCR (all)
*Campylobacter jejuni*	5 (7)	Serum IgM (all)
Zika virus	3 (4)	CSF RT-qPCR (1), CSF IgM (1), and serum IgM (1)
Flavivirus^c^	2 (3)	CSF IgM (all)
Varicella-zoster virus	2 (3)	CSF PCR (all)
Enterovirus	1 (1)	CSF RT-PCR
Not determined^*d*^	33 (45)	-
Total	74 (100)	

CSF: cerebrospinal fluid; PCR: polymerase chain reaction; RT-PCR: reverse transcription polymerase chain reaction; RT-qPCR: quantitative reverse transcription real-time polymerase chain reaction; *a*: encephalitis, transverse myelitis, encephalomyelitis, and Guillain-Barré syndrome; *b*: the arboviral neuroinvasive diseases were classified as confirmed if CSF or serum RT-PCR tests resulted positive or CSF IgM was detectable, and as probable if only serum IgM was detectable; *c*: simultaneous detection of IgM against dengue virus and Zika virus; *d*: negative serological and molecular tests for arboviruses, enteroviruses, herpesviruses and *Campylobacter jejuni*.

Laboratory indicators for current (by RT-qPCR) or recent (by IgM-ELISA) infection by DENV, ZIKV or CHIKV in serum or CSF were found in 26 (35%) of the neurological cases reported, including four deaths (Table I). According to CDC laboratory case definitions for arboviral neuroinvasive diseases, 15 suspected cases were classified as probable and 11 as confirmed (Table II). The surveillance strategy also detected cases attributable to herpes simplex virus, varicella-zoster virus and enteroviruses (Table I). There were no cases attributed to *Flavivirus* other than DENV and ZIKV, to *Alphavirus* other than CHIKV, or to *Orthobunyavirus*. During the assessed period, 7,512 cases of dengue, chikungunya or Zika fever were reported in Piauí state, resulting in a proportion of three neuroinvasive cases for every 1,000 arboviral disease reports.^8^

Neurological cases attributed to arboviruses were associated with fever (p = 0.01) and myalgia (p = 0.04), but an association between these cases and cutaneous rash, conjunctival hyperaemia, arthralgia, arthritis, leukopenia or thrombocytopaenia was not found. The diagnosis of recent arboviral infection was not related to a greater occurrence of intra-hospital death (Table III).

## DISCUSSION

The preliminary results of the surveillance protocol for neurological manifestations likely associated with viral infections in Piauí state showed that around one third of the infectious, post-infectious or parainfectious neurological syndromes reported in the first 12 months after its implementation could have been related to infection by DENV, CHIKV or ZIKV.

Before the emergence of the Zika and chikungunya viruses and the implementation of sentinel surveillance for neuroinvasive infectious syndromes, a similar study completed in a city in the Brazilian Amazon also showed a participation of arboviruses in the genesis of neurological cases.^20^ The World Health Organization has considered the presence of neurological manifestations as a marker of severe dengue presentation since 2009. A study conducted in Rio de Janeiro state, Brazil, showed that DENV infection was responsible for nearly 50% of hospitalisations for meningoencephalitis, from 2006 to 2008.^21^

Neurological complications account for up to 25% of atypical cases and up to 60% of severe atypical cases of CHIKV infection. Reported cases range from encephalitis, optic neuritis, or myeloradiculitis to GBS.^22^ In many cases, neurological signs start after a symptom-free interval of one-three weeks, pointing to an autoimmune process.^3^ ^,^ ^22^

One single-centre Brazilian cohort showed that ZIKV appeared to be associated with an increase in the incidence of a spectrum of serious neurologic syndromes among adults.^23^ A case-control study indicated that GBS was strongly associated with Zika-like illness in Bahia state, Brazil, suggesting that acquired ZIKV infection may result in severe neurologic complications.^24^ Our study investigated the arboviral aetiology in a series of neurological cases during a period when dengue, Zika and chikungunya arboviruses were circulating in Piauí state, Brazil.

The prevalence of laboratory indicators of recent or current infection by arboviruses among the neurological cases registered during the studied period contrasts with previous descriptions from non-tropical countries, where herpesviruses and enteroviruses are involved in the genesis of the most CNS viral infections. The current guidelines for encephalitis management estimates that CSF PCR tests for HSV-1, HSV-2, varicella-zoster virus and enteroviruses can identify 90% of the cases due to known viral pathogens.^25^ ^,^ ^26^ ^,^ ^27^ Now, amid the concurrent epidemics of DENV, ZIKV and CHIKV, this projection cannot be applied to Brazil or, possibly, to other tropical countries because a large proportion of the population is exposed to the *Aedes aegypti* and to these three arboviruses. In addition, there is evidence of other neurotropic arboviruses circulating in Brazil, such as WNV, St. Louis encephalitis virus, Ilheus virus, and Oropouche virus.^7^ ^,^ ^9^ However, the large percentages of arboviral cases found in this study may reflect physicians’ practices of referring cases of encephalitis and other neurological syndromes that are clinically more probable to be attributed to DENV, ZIKV, or CHIKV.

Surveys conducted in non-tropical countries have shown that respiratory and gastrointestinal infections are the main early triggers for GBS. The most frequently identified cause of GBS preceding infection worldwide is the Gram-negative bacillus *C. jejuni*.^28^ However, in Brazil and other tropical countries, DENV, ZIKV or CHIKV certainly play a more important role in GBS genesis than previously described.

The only clinical findings suggestive of arboviral infection associated with laboratory diagnosis of DENV, ZIKV and CHIKV in patients with neurological manifestations in this study were fever and myalgia. However, accurate information about symptoms in patients with encephalitis that quickly evolved to aphasia or coma was limited. In addition to this, there is an extensive overlap between classic arboviral clinical manifestations and infections by other neurotropic viruses. For example, exanthema could occur in infections caused by enteroviruses or by varicella-zoster virus, and conjunctival hyperaemia occurs in infections caused by human herpesvirus I virus and enterovirus.^27^ In addition, the excessive percentage of neurological cases with undetermined aetiology may have hampered the precision of association inferences. Despite the use of advanced microbiological diagnostic tools, aetiological diagnosis is reached in less than 50% of cases of presumed viral infections of the CNS^25^ ^,^ ^26^ - consistent with our study.

Occurrence of leukopenia and thrombocytopenia is rarely reported in ZIKV or CHIKV infection cases.^29^ These cases were not associated with a greater occurrence of intra-hospital deaths, in contrast to herpetic encephalitis, which leads to high morbidity and mortality rates, even among patients treated with acyclovir.^26^ ^,^ ^27^

**TABLE II:** Laboratory test results and CDC classification of cases of arboviral neuroinvasive diseases notified in Piauí state, 2015-2016

Arboviral etiology	Case enrollment	Clinical syndrome	Acute serum												Convalescent serum					CSF tests												CDC neuroinvasive arboviral disease classification
			Collection date	ELISA-IgM tests						PCR tests^*c*^					ELISA-IgM tests					Collection date	ELISA-IgM tests					PCR tests						
				DENV	ZIKV	WNV	CHIKV	*C. jejuni* ^*b*^		DENV	ZIKV	WNV	CHIKV		DENV	ZIKV	WNV	CHIKV			DENV	ZIKV	WNV	CHIKV		DENV^*d*^	ZIKV^*d*^	WNV^*d*^	CHIKV^*d*^	Others^*e*^		
DENV	1	ATM	Day 90	+ve	-ve	-ve	-ve	nd		nd	nd	nd	nd		na	na	na	na		na	na	na	na	na		na	na	na	na	na		Probable
	2	Encephalitis	Day 13	+ve	-ve	-ve	-ve	nd		-ve	-ve	-ve	-ve		na	na	na	na		Day 11	-ve	-ve	-ve	-ve		-ve	-ve	-ve	-ve	HSV, VZV and EV		Probable
	3	Encephalitis	Day 4	-ve	-ve	-ve	-ve	nd		+ve	-ve	-ve	na		na	na	na	na		Day 4	-ve	-ve	nd	nd		+ve	-ve	-ve	na	HSV, VZV and EV		Confirmed
	4	Encephalitis	Day 11	-ve	-ve	-ve	-ve	nd		na	na	na	na		+ve	-ve	-ve	-ve		Day 10	-ve	-ve	-ve	-ve		+ve	-ve	-ve	-ve	HSV, VZV and EV		Confirmed
	5	Encephalitis	Day 14	+ve	-ve	-ve	-ve	nd		-ve	-ve	-ve	na		na	na	na	na		Day 13	-ve	-ve	na	-ve		-ve	-ve	-ve	na	HSV, VZV and EV		Probable
	6	GBS	Day 3	+ve	-ve	-ve	-ve	-ve		-ve	-ve	-ve	na		na	na	na	na		Day 3	-ve	-ve	-ve	-ve		-ve	-ve	-ve	-ve	nd		Probable
	7	GBS	Day 13	+ve	-ve	-ve	-ve	-ve		nd	nd	nd	nd		na	na	na	na		na	na	na	na	na		na	na	na	na	na		Probable
	8	GBS	Day 6	+ve	-ve	-ve	-ve	-ve		-ve	-ve	nd	-ve		na	na	na	na		Day 8	-ve	-ve	-ve	-ve		-ve	-ve	-ve	-ve	nd		Probable
ZIKV	9	ATM	Day 8	-ve	+ve	-ve	-ve	nd		nd	nd	nd	nd		-ve	+ve	-ve	-ve		Day 8	na	+ve	-ve	na		-ve	-ve	na	-ve	HSV, EV		Confirmed
	10	Encephalitis	Day 34	-ve	+ve	-ve	-ve	nd		nd	nd	nd	nd		na	na	na	na		Day 33	-ve	-ve	-ve	na		na	na	na	na	HSV, VZV and EV		Probable
	11	Encephalitis	Day 6	-ve	-ve	-ve	-ve	nd		-ve	+ve	-ve	-ve		na	na	na	na		Day 5	-ve	-ve	-ve	-ve		-ve	+ve	-ve	-ve	HSV, VZV and EV		Confirmed
Flavivirus	12	GBS	Day 5	+ve	+ve	-ve	-ve	-ve		na	-ve	na	na		na	na	na	na		Day 4	+ve	+ve	-ve	-ve		-ve	-ve	-ve	-ve	nd		Confirmed
	13	GBS	Day 8	+ve	+ve	-ve	-ve	-ve		nd	nd	nd	nd		na	na	na	na		Day 8	+ve	+ve	-ve	na		-ve	-ve	na	-ve	nd		Confirmed
CHIKV	14	ADEM	Day 23	-ve	-ve	-ve	+ve	nd		nd	nd	nd	nd		-ve	-ve	-ve	+ve		na	na	na	na	na		na	na	na	na	na		Probable
	15	ATM	Day 10	-ve	-ve	-ve	+ve	nd		nd	nd	nd	nd		-ve	-ve	-ve	+ve		Day 10	-ve	-ve	-ve	+ve		na	na	na	na	HSV, VZV, EV		Confirmed
	16	Encephalitis	Day 14	-ve	-ve	-ve	+ve	nd		nd	nd	nd	nd		na	na	na	na		Day 14	-ve	na	-ve	+ve		na	na	na	-ve	HSV, VZV		Confirmed
	17	Encephalitis	Day 54	-ve	na	-ve	+ve	nd		nd	nd	nd	nd		-ve	-ve	-ve	+ve		Day 53	-ve	-ve	-ve	+ve		nd	nd	nd	nd	HSV, VZV, EV		Confirmed
	18	Encephalitis	Day 8	-ve	-ve	-ve	+ve	nd		-ve	-ve	-ve	-ve		-ve	-ve	-ve	+ve		Day 8	-ve	-ve	-ve	+ve		-ve	-ve	-ve	-ve	HSV, VZV, EV		Confirmed
	19	Encephalitis	Day 25	-ve	na	-ve	+ve	nd		nd	nd	nd	nd		-ve	-ve	-ve	-ve		Day 23	na	na	na	na		na	na	na	na	HSV, VZV, EV		Probable
	20	Encephalitis	Day 14	-ve	-ve	-ve	+ve	nd		nd	nd	nd	nd		na	na	na	na		na	na	na	na	na		na	na	na	na	na		Probable
	21	Encephalitis	Day 36	-ve	-ve	-ve	+ve	nd		nd	nd	nd	nd		na	na	na	na		Day 35	-ve	-ve	na	-ve		nd	nd	nd	nd	HSV, VZV		Probable
	22	Encephalitis	Day 8	-ve	-ve	-ve	+ve	nd		nd	nd	nd	+ve		na	na	na	+ve		Day 7	na	na	na	na		na	na	na	na	HSV, VZV, EV		Confirmed
	23	Encephalitis	Day 15	-ve	-ve	na	+ve	nd		-ve	-ve	-ve	-ve		na	na	na	na		Day 13	-ve	-ve	na	na		-ve	-ve	-ve	-ve	HSV, VZV, EV		Probable
	24	Encephalitis	Day 8	-ve	-ve	-ve	+ve	nd		-ve	-ve	-ve	-ve		na	na	na	na		Day 7	-ve	na	na	-ve		-ve	-ve	-ve	na	HSV, VZV, EV		Probable
	25	Encephalitis	Day 8	-ve	-ve	-ve	+ve	nd		-ve	-ve	-ve	-ve		-ve	-ve	-ve	+ve		Day 8	-ve	-ve	-ve	-ve		-ve	-ve	-ve	-ve	HSV, VZV, EV		Probable
	26	Encephalitis	Day 11	-ve	-ve	na	-ve	nd		-ve	-ve	-ve	-ve		-ve	-ve	-ve	+ve		Day 9	-ve	-ve	na	-ve		-ve	-ve	-ve	na	HSV, VZV, EV		Probable

+ve: positive; -ve: negative; *a*: after symptoms onset; *b*: performed only on GBS patients samples; *c*: performed routinely on samples collected up to the fifth day of symptom onset; *d*: performed routinely on samples collected up to the fifteenth day of symptom onset; *e*: not done on GBS patients samples; all negative; ADEM: acute disseminated encephalomyelitis; ATM: acute transverse myelitis; CHIKV: chikungunya virus; DENV: dengue virus; ELISA: enzyme-linked immunosorbent assay; EV: enterovirus; GBS: Guillain-Barré syndrome; HSV: herpes simplex virus; na: sample not available or inadequate volume; nd: not done; PCR: polymerase chain reaction; VZV: Varicella-Zoster virus; WNV: West Nile virus; ZIKV: Zika virus.

The results of the sentinel surveillance in Piauí state should be interpreted with caution since many cases were diagnosed only by detection of serum IgM. Indeed, there is an extensive cross-reactivity between flaviviruses, and IgM remains detectable in the serum for approximately three months after the infection. Therefore, although unlikely, it is possible that the serum IgM-ELISA results were due to previous and unrelated infections by the tested arboviruses or by other cross-reactive arboviruses. In fact, CDC case definitions for arboviral neuroinvasive diseases consider as probable the cases where IgM antibodies are detected only in serum and, as confirmed, the cases where IgM is detected in CSF or the viral genome is detected in CSF or serum. Although the detection of neutralising serum antibodies allied to IgM reactivity can also confirm neuroinvasive arboviral infection, the plaque reduction neutralisation test (PRNT) is not always able to provide a definitive determination of the specific flavivirus causing a recent infection, particularly in people with a prior history of flavivirus infection. For this reason, PRNT confirmation is not routinely recommended for people living in areas with high levels of circulating flaviviruses.^19^ Here, just over half of the neuroinvasive arboviral diseases were classified as probable. However, the cases in which there was simultaneous positivity for ZIKV and DENV by IgM-ELISAs were considered only as presumptive diagnoses of recent infection by *Flavivirus*.^30^

**TABLE III:** Clinical and laboratory findings and in-hospital outcome of 74 patients with possible acute viral neurological impairment^*a*^ registered in the state of Piauí

	Arboviral neuroinvasive disease^*b*^ (n = 26)	Non-arboviral neuroinvasive disease (n = 48)	
	nº (%)	nº (%)	p^*c*^
Clinical findings			
Fever	22 (85)	21 (44)	0.01
Rash	8 (31)	9 (19)	0.19
Conjunctival hyperaemia	2 (8)	4 (8)	0.64
Arthralgia / arthritis	8 (31)	8 (17)	0.14
Myalgia	11 (42)	10 (21)	0.04
Laboratory data			
Leukopenia	1 (4)	3 (6)	0.55
Thrombocytopenia	3 (16)	3 (6)	0.51
In-hospital outcome			
Death	7 (27)	6 (13)	0.11

*a*: encephalitis, transverse myelitis, encephalomyelitis, and Guillain-Barré syndrome; *b*: dengue, Zika or chikungunya fever; *c*: Fisher’s exact test.

In conclusion, the results of the first year of the MoH sentinel surveillance of neurological manifestations that likely are associated to viral infections in Piauí state, Brazil, showed that arboviral infections by DENV, ZIKV and CHIKV played an important role as causes of GBS, encephalitis, and transverse myelitis and reinforce the need for surveillance and systematic investigation of these neurological diseases.
